# Hospital Physicians' Influence on Gastrointestinal Protection during Treatment with Non-Steroidal Anti-Inflammatory Drugs and Acetylsalicylic Acid and the Impact on Prescribing in Primary Care

**DOI:** 10.1371/journal.pone.0081845

**Published:** 2013-12-06

**Authors:** Michael Due Larsen, Jesper Hallas

**Affiliations:** Clinical Pharmacology, University of Southern Denmark, Odense M, Denmark; University of Modena & Reggio Emilia, Italy

## Abstract

**Background:**

The aim of this study was to describe the use of gastrointestinal (GI) protection before, during and after hospitalisation for elderly patients using NSAID or low-dose ASA.

**Methods:**

This study included all elderly patients (75+) admitted to hospital in the period of 1^st^ April 2010 to 31^st^ March 2011 at Odense University Hospital, Denmark, who were regular users of NSAID or low-dose ASA before hospital admission, or had one of these drugs initiated during hospital stay. By using pharmacy dispensing data and a hospital-based pharmacoepidemiological database, the treatment strategy for the individual patients was followed across hospital stay.

**Results:**

In total, 3,587 patients were included. Before hospital admission, 93 of 245 NSAID users (38.0%) and 597 of 1994 user of low-dose ASA (29.9%) had used GI protection. During hospital stay, use of GI protection increased to 75% and 33.9%, respectively. When hospital physicians initiated new treatment with NSAID or with low-dose ASA, 305 of 555 (55.0%) and 647 of 961 (67.3%) were initiated without concomitant use of GI protection. When hospital physicians initiated GI protection, 26.8–51.0% were continued in primary care after discharge.

**Conclusions:**

During hospital stay, the use of GI protection increases, but when new treatment with NSAIDs or low-dose ASA is initiated in hospital, the use of gastrointestinal protection is low. The low use of GI protection is carried on in primary care after discharge.

## Introduction

The association between non-steroidal anti-inflammatory drugs (NSAID) and aspirin/acetylsalicylic acid (ASA) and risk of major gastrointestinal events, including symptomatic peptic ulcers and peptic ulcer complications is well documented [1]–[3]. NSAIDs and low-dose ASA are among the most frequently prescribed drugs and up to one third of individuals aged over 65 years have been reported to use NSAID on a daily basis [4]–[6]. Certain risk factors are associated with an increased risk of gastrointestinal complications while taking NSAIDs and low-dose ASA: A history of peptic ulcer, Helicobacter Pylori infection and concurrent use of corticosteroids, SSRI or antithrombotic drugs [7], [8]. Advanced age is a very important risk factor as well [2], [9].

Gastrointestinal complications in elderly patients treated with NSAID and low-dose ASA can be prevented. Regarding NSAID, discontinuation of therapy is first choice of recommendation, and if NSAID cannot be discontinued second choice is additional use of proton pump inhibitors (PPI) or misoprostol [10], [11]. Alternatively, H_2_-receptor antagonists (H2RA) can be used for gastrointestinal (GI) protection, although H2RA are not as effective as PPI [11], [12]. PPIs are also recommended as GI protection for elderly patients treated with low-dose ASA for secondary prevention of cardiovascular disease. Guidelines recommend that high age, concomitant use of corticosteroids, SSRI, antiplatelet therapy and previous GI complications should lead to discontinuation or to use of GI protection [13]–[18]. The specific age above which GI protection should be mandatory in elderly patients is not specified in any guidelines [13], [15]–[17].

Only few studies have been carried out describing the interaction between prescribing patterns in primary and secondary care, by following the patients' medication from general practice to hospital and back again to general practice [19]–[21]. The aim of this study was to describe use of GI protection according to national clinical guidelines in elderly hospitalised patients using NSAID or low-dose ASA, and to describe the influence of hospital physicians' prescribing behaviour on the patients' use of GI protection.

## Materials and Methods

In the present study, we identified and followed all elderly (75+ years) patients who were hospitalised for more than two days in the period of 1 April 2010 to 31 March 2011 and who were regular user of NSAID or low-dose ASA before admission to hospital. In the same time period, we identified all patients (75+ years) who had NSAID or low-dose ASA initiated during hospital stay. These two groups of patients were analysed according to their use of GI protection.

**Figure 1:**
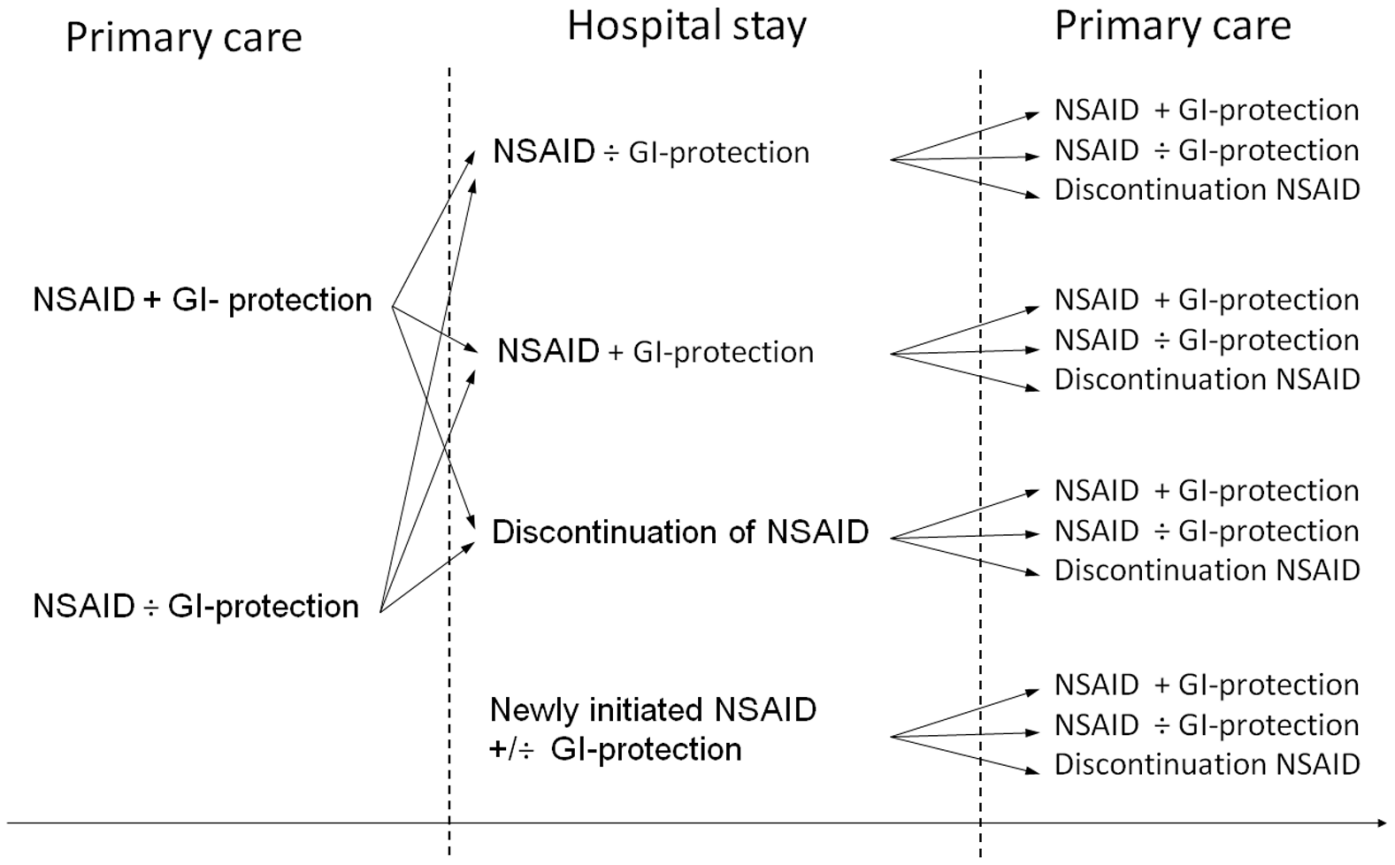
The longitudinal flow for patients treated with NSAID and the use of GI protection (PPI, misoprostol or H2RA).

By using pharmacy dispensing data and a hospital-based pharmacoepidemiological database, we followed the medication regimens of the individual patients across a hospital stay at Odense University Hospital, Denmark. The medication regimen for the each patient was compared at three cross-sections: 1) Before hospitalisation, 2) at discharge from hospital and 3) after hospitalisation (Figure 1).

### Setting

The data for this study were retrieved from three registers: Odense University Pharmacoepidemiological Database (OPED), Odense University Hospital Pharmacoepidemiological Database (OUHPED) and Funen County Patient Administrative System (FPAS). All residents in the County of Funen (population 485,000) have had their hospital contacts and diagnoses registered in FPAS since 1977 for inpatient visits and since 1989 for outpatient visits. Diagnoses are coded according to the International Classification of Disease, ICD-8 until January 1994 and thereafter ICD-10.

OPED contains information on reimbursed drugs dispensed in Funen County. Each prescription record accounts for date of dispensing, drug name, quantity and formulation of the drug. Over-the-counter drugs and drugs not reimbursed are not recorded in the database. The OPED database is described in detail elsewhere [22].

The OUHPED database contains information on patient medication at Odense University Hospital (OUH). OUH is a tertiary teaching hospital accommodating 1,300 beds and comprises one of three main centres in the Danish hospital service. The OUHPED database is generated electronically from the hospital's medical record system, Cosmic, and describes medications including date of dispensing, name, quantity and route of administration during hospitalisation. Data on medication are available back to 2009 for all departments at OUH. Patient administrative data and information on diagnoses can be linked to OUHPED by use of a mutual patient-identifier. The database contains a total of 1.6 million prescriptions, covering 105,000 hospitalisations and 900,000 outpatient visits for a total of 150,000 individual patients.

The databases record drugs according to the anatomical-therapeutic-chemical system (ATC) of the World Health Organization, and the dispensed quantities are expressed by the Defined Daily Dose (DDD) methodology [23]. Diagnoses in FPAS are coded according to the International Classification of Disease, 10^th^ revision (ICD-10 code).

The individual patient was followed by using a unique person-identifier (CPR code) from the Danish Civil Registration System which is shared with other health-related registries in Denmark, thereby allowing record-linkage studies [24], [25].

### Identification of current users of NSAID and low-dose ASA

In an attempt to only include subjects with an ongoing use of NSAID and low-dose ASA at hospital admission, we used the following definitions for inclusion [26], [27]: Ongoing use of low-dose ASA (ATC code B01AC06 and B01AC30): The patient was required to have redeemed prescriptions for at least 80% of the days within a 120-day period before hospitalisation, corresponding to at least 96 dose units (of either 50, 75, 100 or 150 mg). Ongoing use of NSAID (ATC code M01A excluding M01AX05): The patient was required to have redeemed prescriptions of an average of 0.5 DDD daily within a 120-day period before hospitalisation, corresponding to 60 DDD altogether. Patients using drugs classified as selective cox-2 inhibitors, celecoxib or etoricoxib, were not included (M01AH01 and M01AH05). Persons who were categorised as users of both NSAID and low-dose ASA (N = 89) were included in both categories in the description.

OUHPED contains a daily updated account of the medication regimen during hospital stay. The use of NSAID, low-dose ASA and other medications for the individual patients was registered on the day of discharge. Incidental use of NSAID and ASA during hospitalisation that was not carried on to the discharge date was not included in the analysis.

### Measuring indicators for use of gastrointestinal protection and carry-over to primary care

During hospital stay, the hospital physicians could choose to continue use of NSAID or low-dose ASA, to discontinue treatment or to add GI protection if not already prescribed. The study was made complex by the number of scenarios the patients could go through before, during or after hospitalisation (Figure 1).

The occurrence of a prescription on a PPI, H2RA or misoprostol during the period of 120 days before hospital admission was used as an indicator for use of GI protection. The prescription should be redeemed before or on the same date as the last redeemed NSAID or low-dose ASA prescription to ensure that the ulcer drug was not initiated due to an ulcer developed after or because of NSAID or ASA initiation.

To describe the drug use after hospitalisation, we used a 120–day window. If a prescription on a drug, e.g. an NSAID, occurred during this time window, the subject was considered a user of that drug after hospitalisation.

To measure the influence of the hospital physicians on drug therapy after discharge in primary care, we calculated a “carry-over” measure. We defined this term as the proportion of changes in drug therapy initiated in hospital (adherence to clinical guidelines or not) which were continued in primary care after discharge. For example, this could be newly initiated PPI or discontinued NSAID and low-dose ASA.

### Data analysis and measures

The use of GI protection was analysed for patients with the following concomitant risk factors: Age; diagnosis of gastrointestinal complications as cause of admission to hospital (ICD-10 code K21, K25-K28); co-medication: Vitamin k-antagonist (ATC-code B01AA), ADP-receptor antagonist (ATC-code B01AC04, B01AC22 or B01AC24); corticosteroids (ATC-code H02AB); selective serotonin reuptake inhibitors (SSRI) (ATC-code N06AB); history of Helicobacter Pylori eradication therapy (ATC-code A02B and J01FA, J01CA or P01AB01) and cardiovascular disease (ICD-10 code I10, I20–I25, I61, I63 or I64 excluding I63.1).

All results were reported using descriptive statistics in exact numbers and with prevalence in percentages. To compare proportions, a z-test with 95% confidence interval was used [28]. All analyses were performed using STATA release 12.

### Ethics

The study was approved by the Danish Data Protection Agency. Approval from an ethics committee was not required according to Danish law.

## Results

Overall, 20,606 patients hospitalised for more than two days were screened for inclusion. In total, 2,150 used NSAID or low-dose ASA before hospital admission, constituting 245 NSAID users and 1,994 low-dose ASA users, respectively. The mean age was 81.2 years (SD: 5.6) and the average duration of hospital stay was 7.3 days. 961 new patients were discharged with use of low-dose ASA, and 555 new patients were discharge with NSAID. For those patients, mean age was 80.9 years (SD: 5.5) and the average duration of hospital stay was 7.8 days.

Among 245 patients using NSAID before admission to hospital 93 (38.0%) used GI protection. The corresponding figure for low-dose ASA was 597 out of 1994 (29.9%) (Table 1). Patients with a history of HP were often users of GI protection (n = 59) (66.3%), as well as patients using corticosteroids (n = 78) (40.4%). Only 31 of all patients had a gastrointestinal diagnosis as cause of the hospital admission. Among those, only 19% used GI protection and this does not differ significantly from the overall result.

**Table 1:** Prevalence rate of concurrent use of GI protection for users of NSAIDs or low-dose ASA before hospital admission, and for new patients starting NSAID or low-dose ASA during hospital stay.

	Users of NSAID or low-dose ASA before admission to hospital			New users of NSAID or low-dose ASA during hospital stay		
	n GI protection proportion% (CI)			n GI protection proportion% (CI)		
Number of individuals	2150			1437		
Sex (male%)	996 (46.3%)			606 (42.2%)		
Age, mean (SD)	81.2 (5.6)			80.9 (5.5)		
Stay in hospital, days (SD)	7.3 (0.2)			7.8 (0.2)		
Users of:						
NSAID	245	93	38.0% (31.9–44.4)	555	250	45.1% (40.9–49.3)
Low-dose ASA	1994	597	29.9% (27.9–32.0)	961	314	32.7% (29.7–35.7)
NSAID + low-dose ASA	89	40	44.9% (34.4–55.9)	243	133	54.7% (48.2–61.1)
Risk groups and co-medication						
Age over 85 years	598	200	33.4% (29.7–37.4)	373	135	36.2% (31.3–41.3)
GI diagnosis	31	6	19.4% (7.5–37.5)	4	3	75.0% (19.4–99.4)
Former helicobacter pylori	89	59	66.3% (55.5–76.0)	47	28	59.6% (44.3–73.6)
Anticoagulant treatment	276	87	31.5% (26.1–37.4)	169	74	43.8% (36.2–51.6)
cCardiovascular disease	604	201	33.3% (29.5–37.2)	373	137	36.7% (31.8–41.9)
Corticosteroids	193	78	40.4% (33.4–47.7)	112	55	49.1% (39.5–58.7)
SSRI	371	134	36.1% (31.2–41.2)	167	69	41.3% (33.8–49.2)

Data are given in numbers, percentages and with 95% confidence intervals unless otherwise indicated.

### Users of NSAIDs

Among the 93 patients using NSAID before admission who also used GI protection, 89.6% used PPI, 9.3% misoprostol and 1.1% H2RA. During hospital stay, 105 (69.1%) of patients without GI protection discontinued their use of NSAID, 9 (5.9%) had GI protection initiated by the hospital physicians and 38 (25%) continued treatment with NSAIDs without GI protection during hospital stay (Table 2). Of the patients using GI protection before hospital admission 57 (61.3%) discontinued NSAID during hospital stay.

**Table 2:** Numbers and prevalence rates of concurrent use of GI protection for users of NSAID and low-dose ASA across hospitalisation.

	All	No modification in hospital			Discontinuation of NSAID/ASA			Initiated GI protection		
	N	Hospital n (%)	Primary care, n (%)		Hospital n (%)	Primary care, n (%)		Hospital n (%)	Primary care, n (%)	
Former users of no GI protection in primary healthcare:										
NSAID	152	38 (25.0%)	→	18 (47.4%)	105 (69.1%)	→	70 (66.7%)	9 (5.9%)	→	3 (33.3%)
Low-dose ASA	1397	923 (66.1%)	→	799 (86.6%)	281 (20.1%)	→	81 (28.8%)	193 (13.8%)	→	111 (57.5%)
Users of any GI protection in primary healthcare:										
NSAID	93	36 (38.7%)	→	15 (41.7%)	57 (61.3%)	→	32 (56.1%)			
Low-dose ASA	597	470 (78.7%)	→	357 (76.0%)	127 (21.3%)	→	43 (33.9%)			

The following continuation of these treatment strategies in primary care is indicated by arrows.

555 new patients received NSAID during hospital stay. Of these, 250 (45.1%) received GI protection (Table 3). After discharge, 416 (75.0%) of these patients did not redeem an NSAID prescription during the following 120 days.

**Table 3:** Numbers and prevalence rates of concurrent use of GI protection for users of NSAID and low-dose ASA treatment initiated by hospital physicians during hospital stay.

	All	+ GI protection			No GI protection			Discontinued NSAID or low-dose ASA
	N	Hospital1 n (%)	Primary care, n (%)		Hospital n (%)	Primary care, n (%)		Primary care n (%)
NSAID	555	250 (45.1%)	→	67 (26.8%)	305 (55.0%)	→	54 (17.7%)	416 (75.0%)
Low-dose ASA	961	314 (32.7%)	→	160 (51.0%)	647 (67.3%)	→	422 (65.2%)	285 (29.7%)

The following continuation of these treatment strategies in primary care is indicated by arrows.

### Users of low-dose ASA

Among the 596 patients using low-dose ASA before hospital admission with concurrent use of GI protection, 98.5% used PPI and 1.5% H2RA, respectively. Out of the 1,397 persons not using GI protection before admission, 193 (13.8%) had GI protection initiated by the hospital physicians, and 281 (20.1%) discontinued their use of low-dose ASA (Table 2). 470 (78.7%) of patients continued treatment with low-dose ASA without GI protection during hospital stay. In total, 408 (20.5%) patients discontinued low-dose ASA in hospital being 127 (21.3%) of the users with GI protection and 281 (20.1%) of the patients without.

961 new patients started treatment with low-dose ASA during hospital stay and of these, 314 (32.7%) had GI protection prescribed (Table 3). After discharge, 285 (29.7%) of these patients did not redeem a prescription for low-dose ASA.

### Carry-over from hospital care into primary care

For patients treated with low-dose ASA and who had GI protection initiated during hospital stay, 111 (57.5%) of initiated GI protection regimes were carried over into primary care (Table 2). For patients who had low-dose ASA discontinued by the hospital physicians, 81 (28.8%) continued without use of low-dose ASA in primary care; in other words 200 (71.2%) restarted low-dose treatment in primary care (Table 2). For patients starting treatment with low-dose ASA and GI protection in hospital, 160 (51.0%) had GI protection carried over into primary care (Table 3).

For patients using NSAID and who had GI protection initiated during hospital stay, 3 (33.3%) of the initiated prescribings were carried over, but figures were very low in that subgroup (Table 2). For patients who had NSAID discontinued by hospital physicians, 70 (66.7%) continued without use of NSAID in primary care (Table 3). For patients who had NSAID and GI protection prescribed during hospital stay, 67 (26.8%) had GI protection carried over into primary care.

## Discussion

Only one third of elderly patients who were regular users of NSAID or low-dose ASA had used GI protection before hospital admission. During hospital stay, this proportion increased, mainly because of discontinuation of NSAIDs or low-dose ASA. This might place hospital physicians in a positive light, were it not for the low proportion of GI protection used for NSAID or low-dose ASA therapies initiated during hospital stay. These findings correspond well with a Dutch study reporting that 40% of patients using NSAID leave hospital without concurrent use of GI protection [21].

The ideal proportion of NSAID and low-dose ASA users who are supposed to also be users of GI protection is not known [13]–[18]. However, this proportion is assumed to be high in our population. The average age was 80 years, and we only included subjects with a more than trivial use of NSAID or low-dose ASA.

Low-dose ASA is the mainstay of long-term prophylactic antiplatelet therapy. We found that one fifth of patients stopped their use of low-dose ASA during hospital stay and 29.7% newly started users discontinued low-dose ASA after discharge. There may be more reasons for this pattern. Firstly, errors in medication lists in the interface between hospital and primary care is a well-documented problem [29], [30]. Furthermore, this may reflect that the low-dose ASA was paused before surgical intervention. Finally, there seems to be some ambiguity about which patients groups should be prescribed low-dose ASA routinely [31].

In Denmark ASA, ibuprofen 200 mg and H_2_RA are available over-the-counter. 3% of ibuprofen and 9% of low-dose ASA are distributed without a prescription [32]. Elderly patients will in most cases have these drugs prescribed by their general practitioner for having their medicine expenses reimbursed, and the OPED database will for the included patients therefore cover almost all drug used.

The utilisation patterns of NSAIDs are fairly unpredictable, reflecting that these drugs are used for acute inflammatory pain and rheumatic conditions with varying intensity. In this study, we aimed to include patients with a regular daily use before admission. This might explain the fairly high proportion of NSAID users who resumed their NSAID after having it discontinued during hospital stay. In hospital, only 45.1% of newly initiated prescribings of NSAID were supplemented with a PPI. One explanation could be that many NSAID treatments are intended for short-term treatment, and thereby the hospital physicians refrain from prescribing a PPI in contrast to recommendations and regardless of the fact that NSAIDs increase the risk of gastrointestinal complications with virtually no latency [1].

Prescription data indicate when patients redeem their prescriptions, but not when the medicine was taken or not [33]. This is a weakness in most register studies, especially regarding NSAIDs for which the erratic pattern of refills reflects the diversity of how patients use NSAIDs [27], [34]. When using an observation window as a proxy for patients' drug exposure, we cannot know whether patients actually take their medication or not. When using a 120-day interval, we favour those with a regular use of NSAID and those who are adherent to their low-dose ASA treatment. If we had chosen a shorter time period or a lower cut-off for the dosage, we would have included more patients with a periodic NSAID, but the degree of adherence to the guideline would probably have been even lower.

In conclusion, we found that many elderly patients admitted to hospital were using NSAID and low-dose ASA without concomitant use of GI protection, and when hospital physicians initiated these treatments, the adherence to the clinical guidelines was at a similarly low level. During hospitalisation, the hospital physicians increased the guideline adherence mainly by discontinuing NSAIDs. The carry-over of GI protection from hospital care into primary care after hospitalisation was at an equally low level. Possibly, more resources should be used to reinforce the guidelines in both sectors and to ensure adequate communication between physicians in primary and secondary care.
